# Effect of Maternal Iron Deficiency Anemia on the Iron Store of Newborns in Ethiopia

**DOI:** 10.1155/2015/808204

**Published:** 2015-02-04

**Authors:** Betelihem Terefe, Asaye Birhanu, Paulos Nigussie, Aster Tsegaye

**Affiliations:** ^1^Department of Hematology and Immunohematology, University of Gondar, Gondar, Ethiopia; ^2^School of Medical Laboratory Science, Addis Ababa University, Addis Ababa, Ethiopia; ^3^Ethiopian Health and Nutrition Research Institute (EHNRI), Addis Ababa, Ethiopia

## Abstract

Iron deficiency anemia among pregnant women is a widespread problem in developing countries including Ethiopia, though its influence on neonatal iron status was inconsistently reported in literature. This cross-sectional study was conducted to compare hematologic profiles and iron status of newborns from mothers with different anemia status and determine correlation between maternal and neonatal hematologic profiles and iron status in Ethiopian context. We included 89 mothers and their respective newborns and performed complete blood count and assessed serum ferritin and C-reactive protein levels from blood samples collected from study participants. Maternal median hemoglobin and serum ferritin levels were 12.2 g/dL and 47.0 ng/mL, respectively. The median hemoglobin and serum ferritin levels for the newborns were 16.2 g/dL and 187.6 ng/mL, respectively. The mothers were classified into two groups based on hemoglobin and serum ferritin levels as iron deficient anemic (IDA) and nonanemic (NA) and newborns of IDA mothers had significantly lower levels of serum ferritin (*P* = 0.017) and hemoglobin concentration (*P* = 0.024). Besides, newborns' ferritin and hemoglobin levels showed significant correlation with maternal hemoglobin (*P* = 0.018; *P* = 0.039) and ferritin (*P* = 0.000; *P* = 0.008) levels. We concluded that maternal IDA may have an effect on the iron stores of newborns.

## 1. Background

Iron deficiency (ID) is the most important cause of nutritional anemia and is the most common micronutrient deficiency worldwide, especially in developing countries [1]. Pregnant women are particularly vulnerable to ID because of the increased metabolic demands imposed by pregnancy involving a growing placenta, fetus, and maternal tissues, coupled with associated dietary risks [2].

In developing countries including Ethiopia, pregnant women commonly begin gestation with depleted or low body iron stores which might make them prone to developing iron deficiency anemia (IDA) [3]. Frequently, the anemia is severe in degree and it coexists with maternal malnutrition [3]. Under these situations, the competing demands of mother and fetus may disturb the normal maternal-fetal iron homeostasis [3–5]. This may have a resultant effect both on the mother and on the fetus, such as premature delivery, intrauterine growth retardation, and neonatal and perinatal death [6]. As the main source of iron for infants until the age of 6 months is the iron endowed from maternal circulation [7], it is logical to question the extension of the effect of maternal IDA on the fetus during and beyond its stay in the womb.

In spite of many researches conducted on this specific issue, consistent findings were not evident. Some have reported the negative impact of maternal IDA on iron stores of newborns [3, 5, 8–11], while others could not find any relationship in between [12–14]. Most of the studies have used serum ferritin as a measurement of iron store, but this serum ferritin has one known drawback as it is an acute phase reactant (APR); it increases during infection, including sub-clinical infections [15]. Therefore in this study, we incorporated another APR that is C-reactive protein (CRP) test to minimize the bias that can be caused due to infection and tried to determine the effect of IDA on the iron store of term newborns.

## 2. Methods 

This study was conducted from December 2011 to February 2012 in Obstetrics and Gynecology Department of St. Paul's hospital, Addis Ababa, Ethiopia. Mothers who had bleeding during pregnancy, preterm delivery (<37 weeks), multiple pregnancy, eclampsia, diabetes mellitus, heart, kidney, lung disease, and hematologic disease were excluded.

A total of 101 mothers and their respective newborns were included first. However, 12 of them were withdrawn from the study because they had anemia other than IDA. Therefore, the final sample comprised 89 mothers and their respective newborns.

Sociodemographic characteristics of study participants were collected using pretested questionnaires and blood samples were collected at the median cubital vein of the mothers during the process of labor and at the placental end of the umbilical cord. Pairs of samples were collected from each mother and cord using K_3_EDTA test tubes (for complete blood counting, CBC) and test tubes with serum gel separator (for ferritin determination and CRP measurement).

CBC and ferritin concentrations were analyzed using Cell-dyn 1800 (Abbott Laboratories, Abbott Park, Illinois) and fully automated Cobas e 411 (Roche Diagnostics GmbH, D-68298 Mannheim, Germany), respectively. CRP was determined by a qualitative slide agglutination test using Cromatest (Linear Chemicals SL, Barcelona, Spain). The instruments were calibrated before the beginning of analyses. Precision test was carried out to assure reproducibility of results provided by the Cell-dyn 1800 analyzer and it was within the acceptable limit stated by the manufacturer. In addition, commercial quality control samples were included in every session of analyses for both CBC enumeration and serum ferritin level determination. Three levels of whole blood controls (high, medium, and low), two levels of plasma control (low, normal), and serum control (positive and negative) were used for CBC ferritin and CRP determinations, respectively. Levy-Jennine (LJ) charts were plotted and the controls were within the 2SD limits with no shifts or drifts detected.

We entered the data from the analyzers and questionnaire into Microsoft Excel and analyzed it using MedCalc Software Version 12.1.4. D'Agostino-Pearson test was used to check the normality of data distribution. Since all of the analytes studied were not normally distributed, nonparametric tests were applied. Frequencies, percentages, medians, and interquartile ranges (IQR) were computed to summarize the data. In order to compare quantitative and qualitative variables between the groups, Mann-Whitney and Chi-square tests were applied, respectively. Association of maternal and newborns parameters were assessed by spearman's correlation. *P* value of <0.05 was considered as statistically significant in all analyses.

The study protocol was approved by the Research Ethics Review Committees of Addis Ababa University and St. Paul's Hospital. In addition, informed verbal consents were collected from the mothers.

## 3. Result

### 3.1. Description of the Sociodemographic and Obstetric Data of Study Participants

We included 89 mothers with their respective newborns. The median age of the mothers was 23 years (IQR = 21–27 years). As clearly presented in Table 1, about one-third of the mothers (34.8%; *n* = 31) had educational level above secondary school, while 29.2% (*n* = 26) of the mothers were illiterates. Housewives were dominant and accounted for 75.3% (*n* = 67) of the participants.

The majority of mothers were primiparous (64.0%; *n* = 57), and also were attending antenatal care (ANC) during their pregnancy. Those mothers who have been taking iron during their pregnancy accounted for 58.4% (*n* = 52) (Table 1).

Most of the babies were delivered through vaginal delivery (78.7%; *n* = 70) and the proportion of male (49.4%; *n* = 44) and female (50.6%; *n* = 45) newborns were almost equal. The babies had median weight of 3100 g (IQR = 2800–3400 g) and a few (12.4%; *n* = 11) had low birth weight (Table 1).

### 3.2. Hematological and Ferritin Status of Mothers and Their Newborns

The median hemoglobin and serum ferritin levels for the mothers were 12.2 g/dL (IQR = 11.3–12.9 g/dL) and 45.5 ng/mL (IQR = 26.8–80.34 ng/mL), respectively (Table 2). The median hemoglobin and serum ferritin levels for the newborns were 16.2 g/dL (IQR = 15.0–17.2 g/dL) and 191.5 ng/mL (IQR = 140.5–264.8 ng/mL), respectively (Table 2). Table 2 also summarizes the median and the IQRs of other studied CBC parameters among mothers and their newborns.

### 3.3. Grouping Study Participants

The mothers were grouped into two categories, NA and IDA based on hemoglobin and serum ferritin concentrations. We used 11 g/dL as cutoff value for maternal hemoglobin concentration after altitude corrections as per World Health Organization (WHO) recommendation [16]. Similarly, the cutoff value for maternal ferritin level was set at 15 ng/mL for those mothers who were not reactive to CRP test and 30 ng/mL for those mothers who were reactive to CRP test in order to balance the effect of infection as recommended by the WHO [15].

Then, mothers showing low hemoglobin concentration (<11 g/dL) and low ferritin level (<15 ng/mL or <30 ng/mL as per their CRP reaction status) were grouped under IDA. Mothers with normal hemoglobin concentration (≥11 g/dL) were classified as NA. Accordingly, 21 mothers (23.6%) were grouped under IDA category while the rest 68 mothers (76.4%) were grouped under NA category. Prevalence of anemia, median differences in hemoglobin, and ferritin levels among newborns of mothers in the two categories were computed and presented in Table 3.

### 3.4. Correlations between Mothers and Newborns Laboratory Parameters

The newborns ferritin level has significant correlation with hemoglobin (*r*_*s*_ = 0.25, *P* = 0.018) and ferritin (*r*_*s*_ = 0.38, *P* < 0.001) levels of their mothers (Table 4). In addition, the newborns hemoglobin had significant correlation with hemoglobin (*r*_*s*_ = 0.22, *P* = 0.039) and ferritin (*r*_*s*_ = 0.28, *P* = 0.008) levels of their mothers (Table 4); additionally the newborns hemoglobin showed significant correlation with mothers mean corpuscular hemoglobin (MCH) and mean corpuscular hemoglobin concentration (MCHC) values (Table 4).

## 4. Discussion

**Figure 1:**
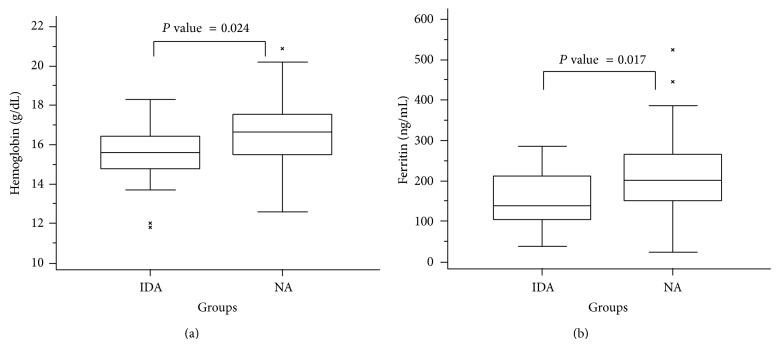
Box plots of hematological profile and ferritin parameters in newborns according to anemia and iron status of the mothers. IDA = iron deficient anemic; NA = nonanemic. *P* values are from the Mann-Whitney test.

In our study, we determined that maternal IDA may have an effect on the iron stores of newborns as hemoglobin (*P* = 0.025) and ferritin concentrations (*P* = 0.027) were significantly lower in newborns delivered from IDA mothers than newborns delivered from NA mothers (Table 3 and Figure 1). These findings were in accordance with previous reports elsewhere [3, 8, 17, 18]. However, there are also findings in contrary to the present study which showed that iron accretion in the fetus was independent of maternal iron status [12–14].

The disagreements might be raised due to differences in cutoff value for serum ferritin (<10 ng/mL, which has low sensitivity), failure to incorporate tests that rule out infection (which may mask the actual ferritin status) [13], and differences in condition of study participants including mothers who were taking iron supplementation during pregnancy, which may have masked the relationship of maternal and newborns iron status [14].

It is well established that serum ferritin is an indicator of the level of body iron sores [19]. Thus, the significantly lower level of ferritin in newborns delivered from IDA mothers compared to NA mothers suggests reduced iron stores in these newborns. Additionally, the newborns delivered from IDA mothers had a significantly lower concentration of hemoglobin than newborns from NA mothers that might contribute for a decreased amount of recycled heme iron resultantly decreasing its contribution for the iron pool. Here, we were not surprised to see no statistically significant difference in prevalence of anemia among newborns of the two groups of mothers (*P* = 0.593). This is because visible difference that can be evidenced in the form of anemia is not expected at such an early stage in life [7]. However, later in life, anemia prevalence could be different among newborns from the two groups of mothers since newborns are highly dependent on the stored iron acquired from the mother during pregnancy till the age of 6 months [20, 21]. Therefore, the significantly lower ferritin level and hemoglobin concentration in newborns delivered from IDA mothers compared to NA mothers may make them prone to iron deficiency and anemia in early infancy. This may have serious consequences on cognitive development and cellular immunity [22].

The evidence presented in this study also denotes that all the hematological and ferritin parameters studied were markedly higher in newborns than in their mothers. Similar findings were also documented in previous studies [3, 5, 8–11]. The higher ferritin levels in newborns can be explained by the existence of active transfer of iron across placenta from mother to the fetus [23]. Also, it can be due to the upregulation of transferrin receptor synthesis in the case of iron deficiency, which enables placenta to compete more effectively for circulating transferrin iron with erythroid marrow of the pregnant mothers intending adequate iron supply of the growing fetus [7, 11, 24].

In this study, newborns ferritin level has significant correlation with hemoglobin and ferritin levels of mothers. In addition, the newborns hemoglobin had significant correlation with hemoglobin and ferritin levels of mothers. Several investigators have determined the correlation between hemoglobin and ferritin parameters of newborns and their mothers; however, the results vary from study to study. Kumar et al., for example, have showed that maternal ferritin levels had significant correlations with Hgb levels (*r*_*s*_ = +0.488; *P* < 0.001) and ferritin (*r*_*s*_ = +0.440; *P* < 0.001) in cord blood [3]. Singla et al. have also found that maternal serum ferritin was significantly correlated with cord blood Hgb (*r*_*s*_ = +0.390, *P* < 0.01) and cord serum ferritin (*r*_*s*_ = +0.523. *P* < 0.001) [8]. The relatively lower correlation observed in this study compared to the two studies may be due to the absence of any severe anemia cases in our study, while there were severe anemia cases in the two studies.

In this study, we determined that the deleterious effect of maternal IDA may extend beyond pregnancy, in an Ethiopian context. This suggests the need for strengthening strategy to improve the maternal iron status. Improving the nutritional status of pregnant women could have a positive impact on improving the iron status of the mothers and also their newborns. The other option might be delayed clamping of the umbilical cord after birth for improving the iron status of young infants [25].

## 5. Conclusion 

Median hemoglobin and ferritin concentrations were significantly lower in newborns delivered from IDA mothers compared to NA mothers. Additionally newborns hemoglobin and ferritin concentration had a significant correlation with hemoglobin and ferritin concentration of the mothers. Based on these findings we can conclude that maternal IDA may have an effect on the iron stores of newborns.

## Figures and Tables

**Table 1:** Summary of sociodemographic and obstetric characteristics of mothers and their newborns gender and weight attending at St. Paul's Hospital, Addis Ababa.

Characteristics	Total (*n* = 89) frequency (%)	IDA (*n* = 21) frequency (%)	NA (*n* = 68) frequency (%)	*P* value^*^
Maternal age				
≤24 yrs	52 (58.4%)	11 (52.4%)	41 (60.3%)	0.700
>24 yrs	37 (41.6%)	10 (47.6%)	27 (39.7%)	
Maternal education level				
No education	26 (29.2%)	6 (28.6%)	20 (29.4%)	0.805
Primary school	19 (21.4%)	6 (28.6%)	13 (19.1%)	
Secondary school	13 (14.6%)	3 (14.2%)	10 (14.7%)	
Above secondary school	31 (34.8%)	6 (28.6%)	25 (36.8%)	
Maternal occupation				
Housewives	67 (75.3%)	16 (76.2%)	51 (75.0%)	0.858
Employed	22 (24.7%)	5 (23.8%)	17 (25.0%)	
Parity				
Primiparous	57 (64.0%)	12 (57.1%)	45 (66.2%)	0.621
Multiparous	32 (36.0%)	9 (42.9%)	23 (33.8%)	
Delivery				
Vaginal	70 (78.7%)	17 (81.0%)	53 (77.9%)	0.992
Cesarean section	19 (21.3%)	4 (19.0%)	15 (22.1%)	
ANC followup				
Yes	79 (88.8%)	17 (81.0%)	62 (91.2%)	0.367
No	10 (11.2%)	4 (19.0%)	6 (8.8%)	
Iron intake during pregnancy				
Yes	52 (58.4%)	10 (47.6%)	42 (61.8%)	0.370
No	37 (41.6%)	11 (52.4%)	26 (38.2%)	
Newborns' gender				
Female	45 (50.6%)	10 (47.6%)	35 (51.5%)	0.953
Male	44 (49.4%)	11 (52.4%)	33 (48.5%)	
Weight of newborns				
Normal birth weight	78 (87.6%)	19 (90.5%)	59 (86.8%)	0.942
Low birth weight	11 (12.4%)	2 (9.5%)	9 (13.2%)	

IDA = iron deficient anemic; NA = nonanemic. ^*^Data are from the Chi-square test.

**Table 2:** Hematological profile and ferritin status of mothers and their newborns at St. Paul's Hospital, Addis Ababa (*n* = 89).

Parameters	Median (IQR)^a^		*P* value^*^
	Mothers	Newborns	
Hemoglobin (g/dL)	12.2 (11.2–12.9)	16.2 (15.0–17.2)	<0.001
Mean cell volume (fL)	90.0 (88.1–93.5)	105.5 (102.7–109.7)	<0.001
Mean cell hemoglobin (pg)	30.7 (30.2–31.8)	37.3 (36.2–38.2)	<0.001
Mean cell hemoglobin concentration (%)	34.2 (33.9–34.8)	35.0 (34.3–35.8)	<0.001
Red cell distribution width (%)	14.1 (13.5–14.9)	16.3 (15.6–17.1)	<0.001
Serum ferritin (ng/mL)	47.0 (26.5–79.7)	187.6 (140.0–264.7)	<0.001

IDA = iron deficient anemic; NA = nonanemic. ^a^IQR, 25th to 75th quartiles, ^*^data are from Mann-Whitney test.

**Table 3:** Hematological profile and ferritin status of newborns by anemia and iron status of their mothers^a^ at St. Paul's Hospital, Addis Ababa (*n* = 89).

Parameters	Group median (IQR)^b^		*P* value
	IDA (*n* = 21)	NA (*n* = 68)	
Hgb (g/dL)	15.6 (14.8–16.4)	16.7 (15.5–17.6)	0.024^*^
MCV (fL)	105.1 (101.6–108.4)	105.9 (103.0–109.9)	0.588^*^
MCH (pg)	37.0 (35.9–38.1)	37.5 (36.4–38.3)	0.344^*^
MCHC (%)	35.0 (33.9–35.4)	35.1 (34.3–35.9)	0.227^*^
RDW (%)	16.0 (15.5–16.5)	16.4 (15.7–17.3)	0.080^*^
Ferritin (ng/mL)	138.9 (105.0–211.7)	200.7 (151.4–265.3)	0.017^*^
Frequency (%) of anemia	3 (14.3%)	5 (7.9%)	0.593^**^

Hgb = hemoglobin; MCV = mean cell volume; MCH = mean cell hemoglobin; MCHC = mean cell hemoglobin concentration; RDW = red cell distribution width. ^a^IDA = iron deficient anemic; NA = nonanemic. ^b^IQR, 25th to 75th quartiles. ^*^Data are from Mann-Whitney test. ^**^Data are from the Chi-square test.

**Table 4:** Spearman's correlation coefficients (*r*) comparing hematological profile and ferritin status of mothers and their respective newborns at St. Paul's Hospital, Addis Ababa (*n* = 89).

Newborns parameters	Mother's parameters *r*_*s*_ (*P* value)					
	Hgb	MCV	MCH	MCHC	RDW	Ferritin
Hgb	0.22^a^	0.09	0.23^a^	0.35^c^	−0.00	0.28^b^
MCV	0.06	−0.03	−0.03	−0.05	−0.08	0.12
MCH	0.15	0.02	0.09	0.14	−0.06	0.10
MCHC	0.16	0.24^a^	0.31^b^	0.39^c^	−0.02	0.04
RDW	−0.01	−0.19	−0.16	0.04	0.03	0.01
Ferritin	0.25^a^	0.10	0.13	0.11	−0.21	0.38^c^

Hgb = hemoglobin; MCV = mean cell volume; MCH = mean cell hemoglobin; MCHC = mean cell hemoglobin concentration; RDW = red cell distribution width. ^a^ *P* value < 0.05, ^b^ *P* value < 0.01, and ^c^ *P* value < 0.001.
